# The *Candida albicans* stress response gene Stomatin-Like Protein 3 is implicated in ROS-induced apoptotic-like death of yeast phase cells

**DOI:** 10.1371/journal.pone.0192250

**Published:** 2018-02-01

**Authors:** Karen A. Conrad, Ronald Rodriguez, Eugenia C. Salcedo, Jason M. Rauceo

**Affiliations:** Department of Sciences, John Jay College of the City University of New York, New York, New York, United States of America; Fred Hutchinson Cancer Research Center, UNITED STATES

## Abstract

The ubiquitous presence of SPFH (Stomatin, Prohibitin, Flotillin, HflK/HflC) proteins in all domains of life suggests that their function would be conserved. However, SPFH functions are diverse with organism-specific attributes. SPFH proteins play critical roles in physiological processes such as mechanosensation and respiration. Here, we characterize the stomatin *ORF19*.*7296/SLP3* in the opportunistic human pathogen *Candida albicans*. Consistent with the localization of stomatin proteins, a Slp3p-Yfp fusion protein formed visible puncta along the plasma membrane. We also visualized Slp3p within the vacuolar lumen. Slp3p primary sequence analyses identified four putative S-palmitoylation sites, which may facilitate membrane localization and are conserved features of stomatins. Plasma membrane insertion sequences are present in mammalian and nematode SPFH proteins, but are absent in Slp3p. Strikingly, Slp3p was present in yeast cells, but was absent in hyphal cells, thus categorizing it as a yeast-phase specific protein. Slp3p membrane fluorescence significantly increased in response to cellular stress caused by plasma membrane, cell wall, oxidative, or osmotic perturbants, implicating *SLP3* as a general stress-response gene. A *slp3Δ/Δ* homozygous null mutant had no detected phenotype when *slp3Δ/Δ* mutants were grown in the presence of a variety of stress agents. Also, we did not observe a defect in ion accumulation, filamentation, endocytosis, vacuolar structure and function, cell wall structure, or cytoskeletal structure. However, *SLP3* over-expression triggered apoptotic-like death following prolonged exposure to oxidative stress or when cells were induced to form hyphae. Our findings reveal the cellular localization of Slp3p, and for the first time associate Slp3p function with the oxidative stress response.

## Introduction

The SPFH protein superfamily is widely conserved throughout all domains of life, but the distribution of SPFH proteins varies in different organisms [1]. Although the structurally conserved SPFH domain is the defining feature, the N- and C- terminal regions are highly divergent [2]. SPFH proteins localize to the plasma membrane and organelle membranes, such as the endoplasmic reticulum, mitochondria, vacuole, and lysosome [2]. SPFH protein functions have been extensively investigated in mammals, nematodes, and several microbes. SPFH proteins have roles in mechanosensation, cell fusion, apoptosis, respiration, morphogenesis, storage, transport, and cell signaling [2–6]. These processes are essential to the pathogenicity of *Candida albicans*, the major fungal pathogen of humans and focus of this study.

Stomatin (STOM), the founding member of SPFH proteins, was first isolated from the plasma membrane of human erythrocytes [7, 8]. The absence of STOM causes a non-lethal hemolytic anemia known as hereditary stomatocytosis [9]. Mutations to the orthologous stomatins MEC-2 in the nematode *Caenorhabditis elegans* and STOML-3 in mice resulted in a loss of sensitivity to mechanical stimuli [10, 11]. Importantly, inhibitors that target STOML-3 may represent a new class of drugs to treat patients with severe nerve injury or diabetic neuropathy and ameliorate acute touch-sensitive pain [12].

Stomatins display both homo- and hetero- oligomerization. The SPFH domain is important for oligomerization, because mutations to SPFH domain sequences abolished homo-oligomerization and plasma membrane localization in mammalian stomatin orthologs [13]. Hetero-oligomeric complexes were observed with human stomatin and various proteins such as the glucose transporter GLUT1, band 3 ion transporter, and the water transporter aquaporin-1 [14]. Also, STOML-3 oligomers interact with Piezo mechanically-gated ion channels in mechanosensation [12]. The diverse functional roles, widespread conservation of SPFH proteins and potential clinical applications underscores the biological significance of the SPFH family.

**Fig 1:**
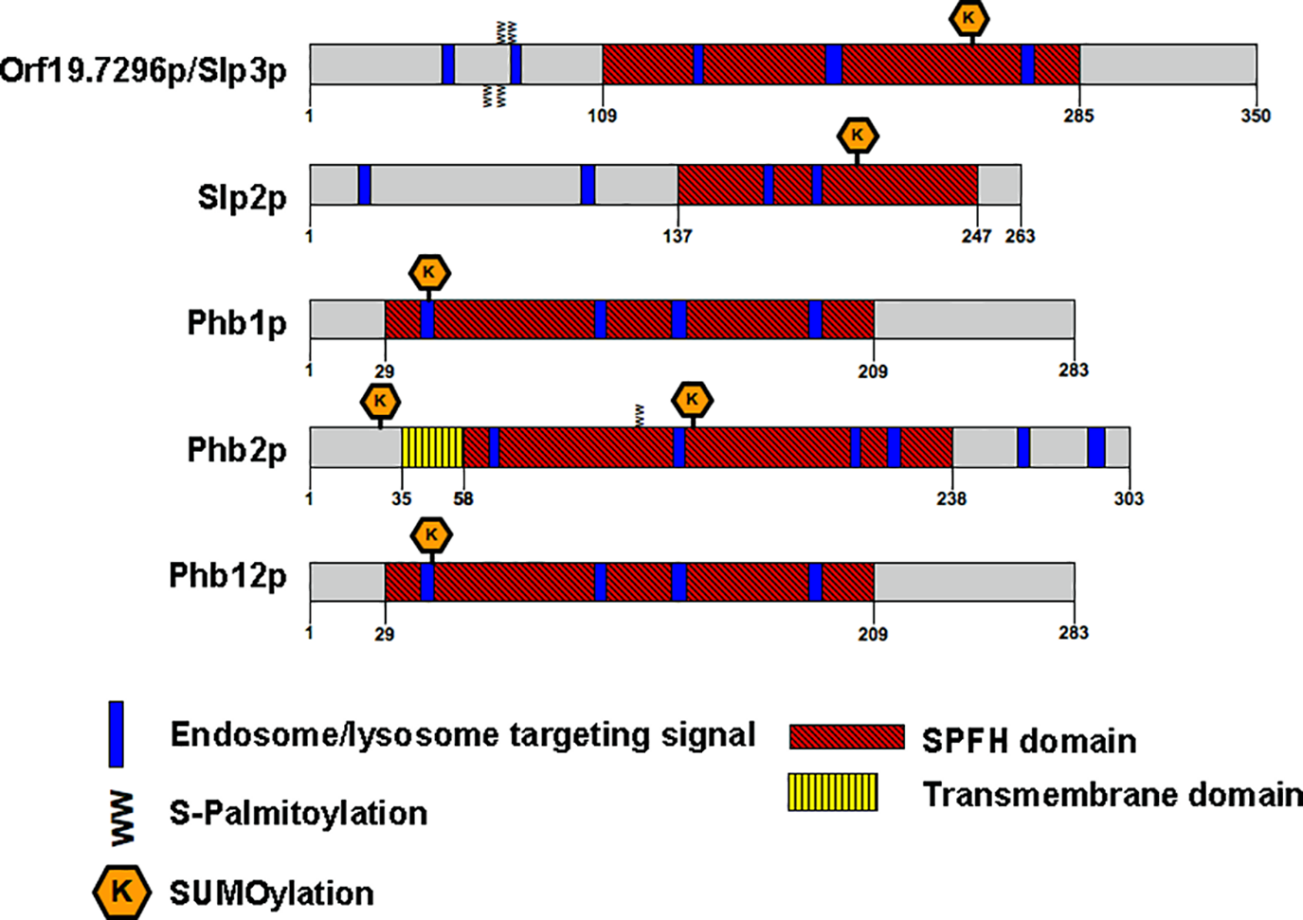
Structure of *C*. *albicans* SPFH proteins. Schematic representation of Orf19.7296p/Slp3p compared to *C*. *albicans* SPFH family members. Images are drawn to scale.

In contrast, SPFH functional information is limited in fungi. In the model yeast *Saccharomyces cerevisiae* the sole SPFH proteins, prohibitin 1 and prohibitin 2 (Phb1p/2p), interacts with Atp23p at the inner mitochondrial membrane to assist formation of ATP synthase [15]. The flotillin FloA, mediates formation of plasma membrane sterol-rich domains, and the stomatin StoA, is required for hyphal polarized growth in the filamentous fungus *Aspergillus nidulans* [6]. The *C*. *albicans* genome includes 5 SPFH family members: *PHB1*, *PHB2*, *PHB12*, *SLP2* (stomatin-like protein 2) and Open Reading Frame (*ORF*) *19*.*7296* (Fig 1). We previously demonstrated that *ORF19*.*7296* is a major target of the High Osmolarity Glycerol (HOG) signaling pathway. *ORF19*.*7296* transcription is significantly increased in cells exposed to acute cationic stress and requires the HOG pathway signaling components Hog1p and Sko1p for full expression [16]. Proteomic analysis implied that Orf19.7296p resides in the plasma membrane [17]. However, Orf19.7296p function remains uncharacterized. More importantly, there is no functional information available for any *C*. *albicans* SPFH proteins.

Here, we utilized a genetic and cellular approach to characterize *ORF19*.*7296*. The Candida Genome Database (CGD) lists the gene name *SLP99* as an alias for *ORF19*.*7296* [18]. However, *SLP99* is also used to describe an unrelated gene. Therefore, we designate *ORF19*.*7296* as *SLP3* (Stomatin Like Protein 3). We show that *SLP3* is a yeast-phase specific general stress response gene. Further, our mutational analysis revealed that Slp3p over-production caused apoptotic-like death when cells are subjected specifically to oxidative stress or when induced to form hyphae.

## Methods

### Yeast strains and media

All *C*. *albicans* strains used in this study were derived from the wild-type (wt) reference strain BWP17 (*ura3*Δ::λ*imm434/ura3*Δ::λ*imm434*, *arg4*::*hisG/arg4*::*hisG*, *his1*::*hisG/his1*::*hisG*) or its derivative strains DAY185 and DAY286 [19, 20]. All strains used in experimental assays were isogenic, and genotypes are listed in Table 1. Cultures were prepared in YPD^+uri^ (1% yeast extract, 2% peptone, 2% dextrose, 80 mg/L uridine) media at 30^°^C with shaking at 225 rpm unless otherwise noted.

**Table 1:** Yeast strains used in this study.

Strain	Genotype
DAY185	*ura3*Δ::λ*imm434/ura3*Δ::λ*imm434*, *ARG*::*URA3*::*arg4*::*hisG/arg4*::*hisG*, *HIS1*::*his1*::*hisG/his1*::*hisG*
ES11	*ura3*Δ::λ*imm434/ura3*Δ::λ*imm434*, *arg4*::*hisG/arg4*::*hisG*, *his1*::*hisG*::*pHIS1/his1*::*hisG*, *slp3*::*URA3/slp3*::*ARG4*
ES26	*ura3*Δ::λ*imm434/ura3*Δ::λ*imm434*, *ARG4*::*URA3*::*arg4*::*hisG/arg4*::*hisG*, *his1*::*hisG/his1*::*hisG*, *SLP3/SLP3-pYFP-HIS1*
JMR249	*ura3*Δ::λ*imm434/ura3*Δ::λ*imm434*, *ARG4*::*URA3*::*arg4*::*hisG/arg4*::*hisG*, *his1*::*hisG/his1*::*hisG*, *SLP3/P*_*TDH3*_*SLP3-pYFP-HIS1*
JMR255	*ura3Δ*::*λimm434/ura3Δ*::*λimm434*, *ARG4*::*URA3*::*arg4*::*hisG/arg4*::*hisG*, *HIS1*::*his1*::*hisG/his1*::*hisG*, *SLP3/P*_*TDH3*_*SLP3*

### Construction of yeast strains

The *SLP3*-yellow fluorescent protein (YFP) tagged strain, *slp3Δ/Δ* homozygous null mutant strain, *P*_*TDH3*_*SLP3*, and *P*_*TDH3*_*SLP3-YFP* over-expressing strains were constructed by PCR-mediated homologous recombination [19, 20]. Oligonucleotide sequences are listed in Table 2. Yeast transformations followed the lithium acetate protocol and were verified by colony PCR [21]. We used plasmid pMG1656 [22] as a template to produce an amplicon containing *YFP*-, *HIS1*-, and *SLP3*- specific sequences. The amplicon was transformed into *C*. *albicans* strain DAY286 and integrated directly following the final amino acid encoding codon to produce strain ES26.

**Table 2:** Oligonucleotide sequences.

Primer Name	Oligonucleotide Sequence 5' - 3'
SLP3FWDPR	AATATTTGAATTTGTGGGGTTTTGATCTGTTGTAGATGCTCATAACTATCAATCAATTATTGCATTGCCTCAATTCGTGTTTTAATTTGTGTAGATACTGTTTCCCAGTCACGACGTT
SLP3REVPR	AGCTATCTGTAGTGCCTAAGAATGCAGACACGTTAACCTTCTCCCAACCTCCATCACCACTTCGAGTACTATTTAAATTGGGTCAAGAGTATCACAAATTGTGGAATTGTGAGCGGATA
SLP3FWDdet	CCGCTCCCAGCTATGTACAA
SLP3REVdet	TCATACGAGCAAATGAACAA
SLP3FWDcomp	TTGA TCAGCCATTT ATTTTCGTT
SLP3REVcomp	TCATACGAGCAAATGAACAA
SLP3/YFPFWDPR	AAGGATTGGGTAGGTACTCCACAAAGTATTGGTATTAACAATAATAGGCATATCAGTGAGACAGTTGCATTGCAAGAAGCCATGAGAGTAGGTGGTGGTTCTAAAGGTGAAGAATTATT
SLP3/YFPRVPR	GGGGACAGGGGTCCACAAAAGATCAAATATCCAACCCAGTTGGAGATGGGGATAACAACTAAGAAACCATTAATA GAATTCCGGAATATTTATGAGAAAC
SLP3OEFWD	ACGTAATTGATCAGCCATTTATTTTCGTTTAAAACATTACCTTTGTGGTACGTGGTATCTCCGAGCTGTATACTGTCAATATCGATAGCAAAGAAGAAAAATCAAGCTTGCCTCGTCCCC
SLP3OERV	TCTCTGGTTTGTGAGTTGGTTGATCGGTAATTGCTGCTTGGGACTTTTTATAAGTATCAGGATCAAAAGAATCAGTGGAATGATTTGATTGAGCTTGCATATTTGAATTCAATTGTGATG
SLP3/OEFwDetPr1	ACGTGGTATCTCCGAGCTGT
SLP3/OERvDetPr2	TCAATGGTGGATCAACTGGA

To construct the *slp3Δ/Δ* homozygous null mutant strain, ES11, both *SLP3* alleles were replaced with *URA3* and *ARG4* via PCR-mediated gene deletion and brought to *HIS* prototrophy through transformation with *Nru*I-digested plasmid pDDB78. To construct the *P*_*TDH3*_*SLP3-YFP* and *P*_*TDH3*_*SLP3* over-expressing strains, JMR249 and JMR255, we used plasmid pCJN542 as a template to produce an amplicon containing the glyceraldehyde-3-phosphate dehydrogenase *(TDH3*) promoter sequences, nourseothricin resistance gene (*NAT*), and *SLP3*-specific sequences. We chose the *TDH3* promoter for over-expression analysis, because it was shown to be constitutively active under *C*. *albicans* invasive conditions [23]. The amplicon was transformed into the *C*. *albicans SLP3-YFP* strain (ES26) and wt strain (DAY 185) and integrated directly upstream of the *SLP3* start codon.

### Microscopy and flow cytometry

Approximately 5 μL of cell suspension was spotted onto poly-lysine coated slides for microscopic analysis. Samples were observed at a total magnification of 1000X with an EVOS compound light microscope (Thermofisher) equipped with the appropriate light cubes for fluorescent visualization. All photographs were processed with ImageJ software. For each assay, three biological replicates were analyzed. Experiments were repeated at least three times unless otherwise noted, and data presented represents one representative experiment.

Fluorescent cells were quantified using an Attune NxT Flow Cytometer (Life Technologies) equipped with a 50 mW, 488 nm LED laser and 530/30 nm emissions filters. Data was analyzed using Attune NxT Software v2.2. Acquisition settings were set to wt untagged control cells by adjusting the voltage to the third logarithmic decade of the fluorescence channel on a histogram plot. We gated out all events with fluorescence below the maximum value for wt untagged control cells to account for potential yeast cell auto-fluorescence, unless otherwise noted. For each assay, three biological replicates were analyzed. Experiments were repeated at least three times, and data presented represents one representative experiment. Ten thousand events were collected for each sample, and the Median Fluorescent Intensity (MFI) was recorded. The average MFI was determined, and statistical differences between samples were analyzed in paired t-tests.

### Slp3p localization assays

*C*. *albicans* cultures were grown overnight for 16 hours in 5 mL YPD^+uri^. Approximately 5.0 x 10^7^ cells were introduced to fresh YPD^+uri^ and incubated for 3–4 hours. Approximately 3.0 x 10^7^ cells were harvested, and resuspended in 100 μL of YPD^+uri^. To visualize Slp3p within the vacuole, samples were incubated with 160 μM of the lipophilic styryl dye FM 4–64 (Life Technologies) and incubated in the dark at room temperature for 40 minutes (pulse). Cells were harvested, resuspended in 1 mL of YPD^+uri^ and incubated for 2.5 hours at 30^°^C (chase). Since FM 4–64 can be used to visualize endosome to vacuolar membrane transport in a time-dependent manner, chase was performed at 15 and 30 minutes to monitor endocytosis and 2.5 hours to ensure only visualization of the vacuole [24]. Cells were washed three times with 1X Phosphate-buffered saline (PBS) solution pH 7.0, resuspended in 1X PBS, and examined under the microscope.

To visualize Slp3p within the plasma membrane, overnight cultures were diluted to an OD_600nm_ of 0.2 in 5 mL of YPD^+uri^ and incubated until an OD_600nm_ of ~0.8–1.0 was obtained. Cells were harvested, washed once with 1X PBS, and 1 x 10^5^ cells were resuspended in 1 mL of 1X PBS. Samples were incubated with 200 μg/mL Filipin III (Sigma) for 5 minutes at room temperature in the dark and examined under the microscope.

To examine Slp3p localization kinetics, overnight cultures were inoculated to an OD_600nm_ of 0.2 in 5 mL of YPD^+uri^ and incubated for the indicated culture time periods. The time points of inoculation were staggered to achieve the indicated culture times and allow for analysis of the samples at the same time. Once all indicated culture time periods were achieved, samples were washed with 1X PBS, diluted to an OD_600nm_ of 1.0 in 1 mL of 1X PBS, and visualized under the microscope. Fluorescent cells were quantified with flow cytometry.

To examine Slp3p localization under environmental stress, cells from overnight cultures were diluted to an OD_600nm_ of 0.2 in 5 mL of YPD^+uri^ and incubated until an OD_600nm_ of ~0.8–1.0 was obtained. Next, cells were treated with the indicated stress agent (or dH_2_O as a control) and incubated for 45 minutes at 30^°^C. Following treatment, samples were immediately examined under the microscope, and fluorescence was quantified with flow cytometry.

To examine Slp3p in the yeast-to-hyphae transition, yeast cells from overnight cultures were harvested and washed three times with 1X PBS buffer. Samples were diluted to an OD_600nm_ of 0.2 in 5 mL of YPD^+uri^ supplemented with 10% Fetal Bovine Serum (FBS) or in Spider medium (1% nutrient broth, 1% mannitol, 11.5 M potassium phosphate, pH 7.2). The cultures were grown for approximately 16–24 hours at 37^°^C, washed three times with 1X PBS, resuspended in 1 mL of PBS, and examined under the microscope. To examine stress-induced localization of Slp3p in hyphal cells, samples were prepared as described above; however, hyphal cells were resuspended in 5 mL of YPD^+uri^ supplemented with 10% FBS and the indicated additive (or dH_2_0 for control). Cultures were incubated at 37^°^C with shaking for 45 minutes followed by microscopic examination.

### Phenotype assays

To examine cytoskeleton structure, cells from overnight cultures were diluted to an OD_600nm_ of 0.2 in YPD^+uri^ and grown for 3 and 16 hours with shaking. Cultures were standardized to an OD_600nm_ of 1.0 and were incubated with 6.6 μM phalloidin rhodamine (Invitrogen) to stain actin filaments as previously described [25]. To visualize tubulin, samples were stained with 150 nM Tubulin Tracker Green (Invitrogen) following manufacturer’s instructions. To visualize the cell wall, cells were stained with 100 μg/mL calcofluor white as previously described [25]. All samples were examined under a microscope as described above.

To examine vacuolar acidification, overnight cultures were standardized to 5.0 x 10^7^ cells/mL in YPD^+uri^ and incubated for 3 hours. Approximately 3.0 x 10^7^ cells were stained with 10 μM 2′,7′-Bis(2-carboxyethyl)-5(6)-carboxyfluorescein acetoxymethyl ester (BCECF AM; Invitrogen) for 40 minutes. Cells were washed three times with 1X PBS and examined under the microscope. To quantitatively examine vacuolar acidification in exponential and stationary phase cultures, the time points of inoculation and sample preparation were performed as described in the Slp3p kinetic localization assays. Samples were quantified using flow cytometry.

Growth assays on solid nutrient growth medium were performed as previously described [26]. Briefly, *C*. *albicans* overnight cultures were diluted to a starting OD_600nm_ of 3.0. Samples were serially diluted, spotted onto designated plates, incubated at 30°C, and photographed after 1–3 days of growth.

To examine growth kinetics under oxidative stress, overnight cultures were standardized to an OD_600nm_ of 0.2 in 200 μL YPD^+uri^ and YPD^+uri^ supplemented with 0.08% sodium dodecyl sulfate (SDS) or 0.17% hydrogen peroxide (H_2_O_2_) in a 96 well plate. Samples were grown for 24 hours with shaking, and the OD_600nm_ was acquired every 30 minutes using a Synergy Mx plate reader (Biotek).

Inductively coupled plasma mass spectrometry was performed to assess intracellular ion levels. *C*. *albicans* cultures were prepared by the procedure outlined in Eide et al [27] with the following adjustments. Overnight cultures of *C*. *albicans* were grown for 16 hours and diluted to an OD_600nm_ of 0.2 in 25 mL YPD^+uri^ media. Samples were incubated with shaking at 30°C to an OD_600nm_ of 1.0. To examine the effects of cation stress, cultures were split and one half was treated with distilled water, while the other was treated with 1.0 M NaCl. The cells were harvested and washed three times in 1 μM EDTA buffer, pH 7.0. Cell pellets were lyophilized overnight at 4°C and the mass recorded. Approximately 100 mg of each sample was acid-digested and analyzed by inductively coupled plasma mass spectrometry at the University of Georgia Center for Isotope Studies. Three biological replicates of each strain were used for each assay, and two independent experiments were performed.

### Apoptosis assays

Overnight cultures were diluted to an OD_600nm_ of 0.2 in 5 mL of YPD^+uri^ supplemented with 0.08% SDS or 0.17% H_2_O_2_ and incubated. Cells were harvested after 3 and 16 hours, washed with 1X PBS, and approximately 1.0 x 10^6^ cells were resuspended in 1 mL of 1X PBS. Samples were treated with 14 μM Propidium Iodide (PI, Thermofisher) and incubated in the dark for 20 minutes at room temperature. Cells were harvested, washed once with 1X PBS, and examined under the microscope. PI-labeled cells were quantified with flow cytometry. We used heat-killed wt cells to establish a profile of inviable cells and used 574/26 nm emissions filters. At least three independent experiments were performed.

To determine the mitochondrial membrane potential, cultures were grown, subjected to oxidative stress, and prepared as described above. Samples were treated with 1X JC-10 (Sigma) following the manufacturer’s protocol. Untreated samples were used as a negative control. We treated wt cells with 10 μM of the mitochondrial membrane perturbant carbonyl cyanide 4-(trifluoromethoxy) phenyl hydrazone (FCCP; ABCAM) as a positive control to profile cells with depolarized mitochondria. JC-10 fluorescence was monitored by flow cytometry using 530/30 nm and 574/26 nm emissions filters.

To determine ROS production, cultures were grown, subjected to oxidative stress, and prepared as described above. Samples were incubated with 5 μg/mL dihydrorhodamine 123 (DHR-123; Sigma) for 10 minutes in the dark at room temperature and then quantitatively analyzed by flow cytometry. Untreated DHR-123-labeled samples were used as controls.

To examine the viability of hyphal cells, samples were prepared as described in the Slp3p localization assays section and treated with 14 μM PI. Samples were visualized under the microscope.

### Slp3p primary sequence analysis

CGD (http://www.candidagenome.org/) was utilized to search for all SPFH family members in *C*. *albicans*. The Aspergillus Genome Database (AspGD, http://www.aspgd.org/) was used to retrieve SPFH sequences from *A*. *nidulans*. Sequences for human, mouse, and nematode SPFH family members were retrieved from UniProt and NCBI: HsSTOM (accession: P27105), HsSTOML1 (accession: Q9UBI4), HsSTOML2 (accession: Q9UJZ1), HsSTOML3 (accession: Q8TAV4), HsPodocin (accession: Q9NP85), MmStoml3 (accession: Q6PE84), CeStomatin2 (accession: NP_001257021.1). Sequences were aligned using T-Coffee [28] with default parameters and then formatted using Boxshade (http://www.ch.embnet.org/software/BOX_form.html). Sequence identities and similarities between Slp3p and other SPFH family members were determined using Protein BLAST with default parameters. Post-translational modification and organelle targeting predictions were made using the bioinformatics resource portal ExPASy (https://www.expasy.org/). Protein schematics were constructed using Illustrator for Biological Sequences, version 1.0 [29].

## Results and discussion

### Primary sequence analysis of Slp3p

*SLP3* was annotated as a *C*. *albicans* stomatin based on homology to the SPFH domain from *Pyrococcus horikoshii* pSTOM [18]. The SPFH domain comprises approximately 50% of Slp3p (Fig 1 [18]). In several mammalian and nematode stomatins, sequences both N- and C-terminal to the SPFH domain are required for membrane insertion, organelle targeting, lipid transport, and contain sites for post-translational modifications [3]. We aligned the full sequence of Slp3p against mammalian, fungal, and nematode stomatins to evaluate the N- and C-terminal regions. We also analyzed the primary sequence of Slp3p for post-translational modification consensus sites and organelle targeting sequences using 8 predictor programs (S1 Table). We did not detect any significant sequence similarities in the N- and C-terminal regions of Slp3p compared to those in mammalian and nematode stomatins (S1 Fig). A short hydrophobic membrane sequence that allows insertion into the plasma membrane with a hairpin-like topology is present in mammalian and nematode stomatins [2]. A highly conserved proline residue within this region is essential for its hairpin-like formation [3]. We did not observe homologous sequences in Slp3p, suggesting that a membrane insertion region may be absent (S1 Fig). We found that the SPFH domain of Slp3p was highly similar to those in mammalian, nematode, and fungal stomatins (55–78% similarity, Table 3). Notably, Trp_184_ is essential for homo-oligomerization in human STOM [30] and is conserved in Slp3p (S1 Fig). We did not identify C-terminal sequences required for sterol binding as observed in human STOML-1 (S1 Fig [31]).

**Table 3:** Summary of primary sequence alignments.

Protein^a^	Entire Sequence^b^			SPFH Domain^c^		
	Identity (%)	Similarity (%)	E-value^d^	Identity (%)	Similarity (%)	E-value^d^
CaSlp3p	100	100	0	100	100	7.00e-131
CaSlp2p	31	53	7.00e-19	34	55	2.00e-16
CaPhb1p	NS	NS	NS	NS	NS	NS
CaPhb2p	NS	NS	NS	NS	NS	NS
CaPhb12p	NS	NS	NS	NS	NS	NS
AnStoA	52	72	7.00e-103	58	78	4.00e-62
AnFloA	NS	NS	NS	NS	NS	NS
HsSTOM	32	59	2.00e-33	36	61	1.00e-24
HsSTOML1	NS	NS	NS	NS	NS	NS
HsSTOML2	32	54	9.00e-27	34	56	3.00e-25
HsSTOML3	34	59	2.00e-35	35	61	3.00e-28
HsPodocin	29	54	9.00e-28	28	58	8.00e-22
MsStoml3	34	59	8.00e-36	35	61	3.00e-28
CeStomatin2	31	56	7.00e-37	34	64	1.00e-35

^a^ This column lists the SPFH family protein from its respective organism. Ca: *Candida albicans*; An: *Aspergillus nidulans*; Hs: *Homo sapiens*; Ms: *Mus musculus*; Ce: *Caenorhabditis elegans*.

^b^This column shows the percent identity, similarity and E-values of Slp3p compared against the listed SPFH family members as determined using Protein BLAST with default parameters.

^c^ This column shows the percent identity, similarity and E-values of the Slp3p SPFH domain exclusively compared against the listed SPFH family members. For fungal SPFH family members, SPFH domain sequences were retrieved from the domain/motifs page on CGD and AspGD, respectively. For mammalian SPFH family members, SPFH domain sequences were retrieved from the family & domains section of each protein on UniProt. For CeStomatin2, the SPFH domain sequence was retrieved from the Features section of the protein on NCBI.

^d^Comparisons that provided an E-value > e^-10^ were considered non-significant (NS) as previously described [5].

Consistent with stomatin orthologs, we identified four putative S-Palmitoylation sites in the N-terminal region at residues Cys^65^, Cys^69^, Cys^70^, and Cys^72^ (Fig 1 and S1 Fig), which may mediate plasma membrane localization and subcellular trafficking [32]. Human stomatin-like protein-1 (SLP-1) contains a GYXXφ motif in the N-terminal region, where X is any amino acid and φ is a bulky hydrophobic residue (GYRAL). This motif targets SLP-1 to late endosomal compartments where SLP-1 controls cholesterol transfer [31]. We found that Slp3p also contains a similar motif (GYQSF) in the N-terminal region and four additional endosomal targeting motifs, suggesting that it may localize to endosomes (Fig 1 and S1 Fig). We also found a potential SUMOylation site that may facilitate membrane targeting and subcellular trafficking (Fig 1 and S1 Fig [33, 34]).

In stomatins, sequences N- and C-terminal to the SPFH domain are usually divergent among family members [3]. We find that this feature is similar in *C*. *albicans* Slp3p. Moreover, our findings show that sequences which govern plasma membrane localization and organelle targeting in mammalian stomatins are conserved in Slp3p.

### Localization of Slp3p

**Fig 2:**
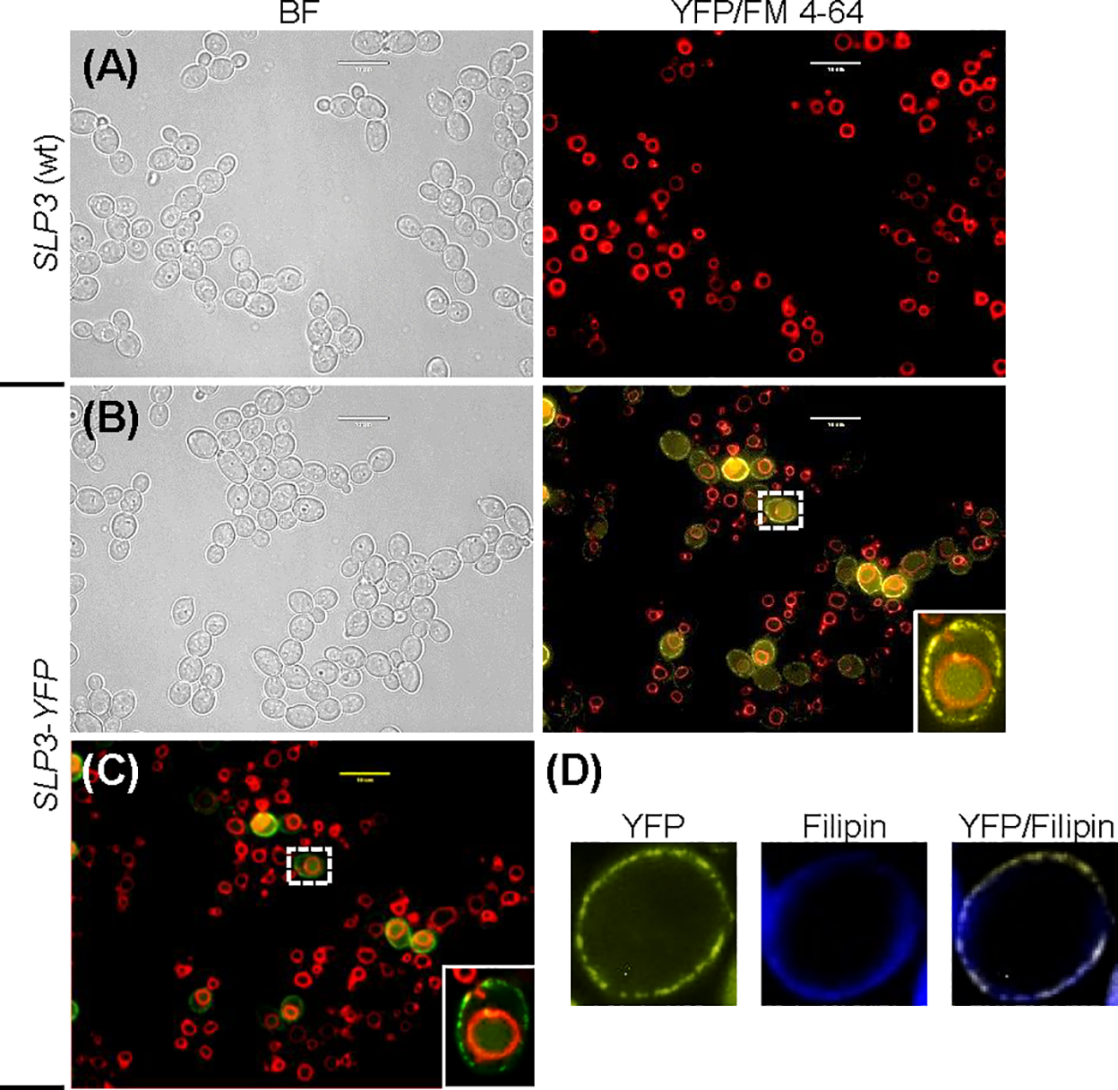
Localization of Slp3p. (A) Exponential-phase samples of the untagged wt *SLP3* control strain and (B) *SLP3-YFP* expressing strain were treated with 160 μM FM 4–64. The FM 4–64 incubation period was set to allow visualization of the vacuole. Cells were viewed under bright-field (BF) and fluorescent microscopy. The right panels show an overlay of the YFP and FM 4–64 fluorescence (YFP/FM 4–64). (C) Overlay photo shown in (B) was recolored with ImageJ to assess Slp3p-Yfp vacuolar membrane localization. The yellow fluorescence color was reassigned to green. (D) Overnight-cultured *SLP3-YFP* cells were treated with 200 μg/mL Filipin and viewed using fluorescent microscopy. Image depicts a single cell, and the right panel shows an overlay of the YFP and Filipin fluorescence (YFP/Filipin). The dashed white box highlights the cell depicted in the inset image. For each assay, three biological replicates were analyzed. Experiments were repeated at least three times, and data presented represents one representative experiment. Approximately 1.0 x 10^4^ cells of each strain were selected for viewing (A-D). Scale bars represent 10 μm.

The presence of palmitoylation sites and the SPFH domain implies that Slp3p resides in the plasma membrane. Stomatins are typically visualized with fluorescent microscopy as punctate foci along the plasma membrane or other organelle membranes [5, 6, 31, 35]. We created a Slp3p-Yfp fusion protein and monitored cellular localization with fluorescence microscopy in exponential-phase cells. Consistent with stomatin family members, Slp3p-Yfp formed distinct puncta along the plasma membrane and co-localized with the membrane sterol-binding dye, Filipin (Fig 2B and 2D). Our Slp3p-Yfp fluorescent findings were not attributed to yeast cellular auto-fluorescence, because untagged wt control *SLP3* cells do not fluoresce (Fig 2A). Also, fluorescence was not observed when cells were viewed using light cubes that select for green, red, and cyan fluorescence (data not shown). In humans, homo-oligomerization of stomatin monomers and/or hetero-oligomerization between stomatins with their respective interacting protein promotes the formation of plasma membrane puncta [14, 36]. The sequence conservation in the Slp3p SPFH domain to human stomatins (61% similarity, Table 3) and plasma membrane puncta strongly suggests that Slp3p forms homo-oligomeric, hetero-oligomeric, or both types of structures in the *C*. *albicans* plasma membrane.

Unexpectedly, we also observed localized regions of intracellular fluorescence in several cells (Fig 2B). We reasoned that the intracellular fluorescence was due to vacuolar accumulation of Slp3p. First, human SLP-1, localizes to late endosomes [31]. In yeast, the endosome fuses with the vacuole during endocytic trafficking. Second, the SLP-1 endosomal localization consensus sequence is conserved in Slp3p (S1 Fig). Third, *A*. *nidulans* StoA is transported in endosomes along hyphae [6]. We treated cells with the vacuolar membrane dye FM 4–64 and found that Slp3p-Yfp localized within the vacuole (Fig 2B). Also, vacuolar localization was consistent in cells containing fragmented vacuoles (S2 Fig). To rule out the possibility that Slp3p resides in the vacuole membrane and lumen, we used ImageJ to recolor yellow fluorescent cells green. If Slp3p resided at both vacuolar sites, then we would expect to see a region of yellow florescence (caused by FM 4–64’s red fluorescence overlaying with green) at the vacuolar membrane. Our recolored image confirmed that Slp3p-Yfp resides in the vacuolar lumen (Fig 2C). The yeast vacuole serves as a site for protein degradation, ion storage, and endomembrane trafficking [37, 38]. Stomatins have been associated with these processes [2], and our fluorescence microscopy findings show for the first time the distribution of Slp3p in the plasma membrane and Slp3p vacuolar localization.

### *SLP3* expression

**Fig 3:**
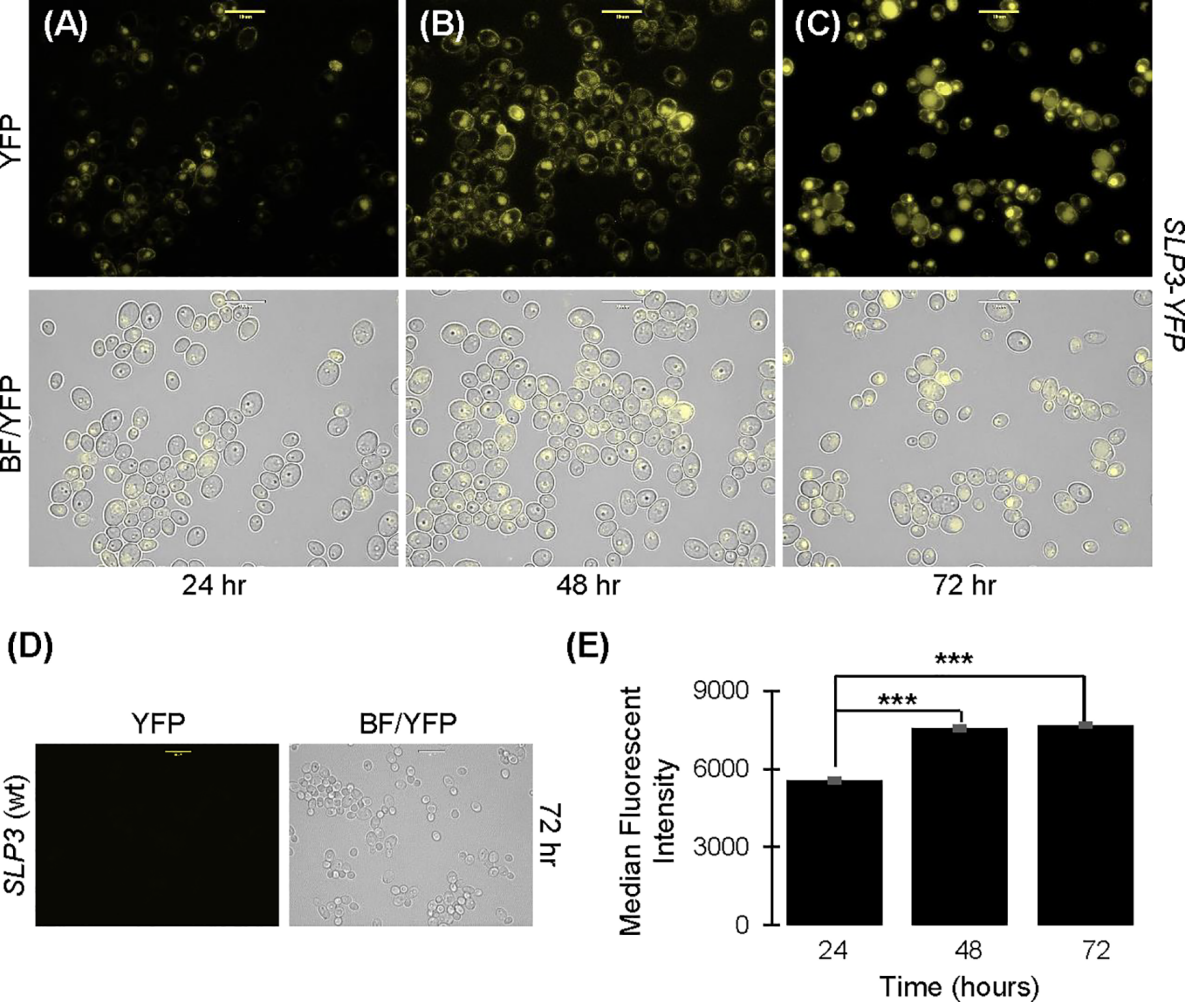
Growth phase localization of Slp3p. Samples of *SLP3-YFP* cells were collected at (A) 24, (B) 48, and (C) 72 hours and examined under bright-field and fluorescent microscopy. (D) Fluorescence of untagged wt *SLP3* control cells grown for 72 hours was examined to assess potential yeast auto-fluorescence. Approximately 1.0 x 10^4^ cells of each strain were selected for viewing (A-D). Scale bars represent 10 μm. (E) Fluorescence of *SLP3-YFP* samples was quantified by flow cytometry at the indicated time points. Untagged wt *SLP3* control cells were analyzed at identical time points and served as a negative fluorescent control. For each assay, three biological replicates were analyzed. Experiments were repeated at least three times, and data presented represents one representative experiment. ***p < 0.001 when compared to *SLP3-YFP* cells grown for 24 hours. Scale bars represent 10 μm.

Unusually, some exponential-phase *SLP3-YFP* cells displayed greater fluorescence than other cells (Fig 2B). We considered that *SLP3* expression may be growth-phase dependent, since *C*. *albicans* grows asynchronously. We monitored cellular fluorescence in early, mid, and late stationary-phase cultures. Our fluorescent images showed that the membrane fluorescence and number of fluorescent cells was greater in stationary-phase cultures compared to logarithmic-phase cultures (compare Figs 2B and 3A–3C). This increase was not attributed to yeast cellular auto-fluorescence from aging cultures, because fluorescence was not observed in the untagged wt *SLP3* control strain after 72 hours of growth (Fig 3D). Fluorescence quantification using flow cytometry showed that the fluorescence intensity in mid and late stationary-phase cultures (48 and 72 hours growth) was statistically greater than early stationary-phase (24 hours growth) cultures (Fig 3E and S1 Appendix). The fluorescence intensity peaked at approximately 48 hours of growth and was unchanged after 72 hours.

*SLP3* is annotated as a putative cation conductance protein [18]. We previously found that *SLP3* transcription significantly increased when cells were briefly exposed to 1.0 M NaCl [16]. Therefore, we wanted to determine whether Slp3p localization increases following exposure specifically to NaCl stress or general cationic stress. We examined cellular fluorescence of *SLP3-YFP* cells subjected to cationic stress caused by transition metals, divalent cations, and monovalent cations. Slp3p-Yfp fluorescence increased under all conditions examined (S3 Fig).

**Fig 4:**
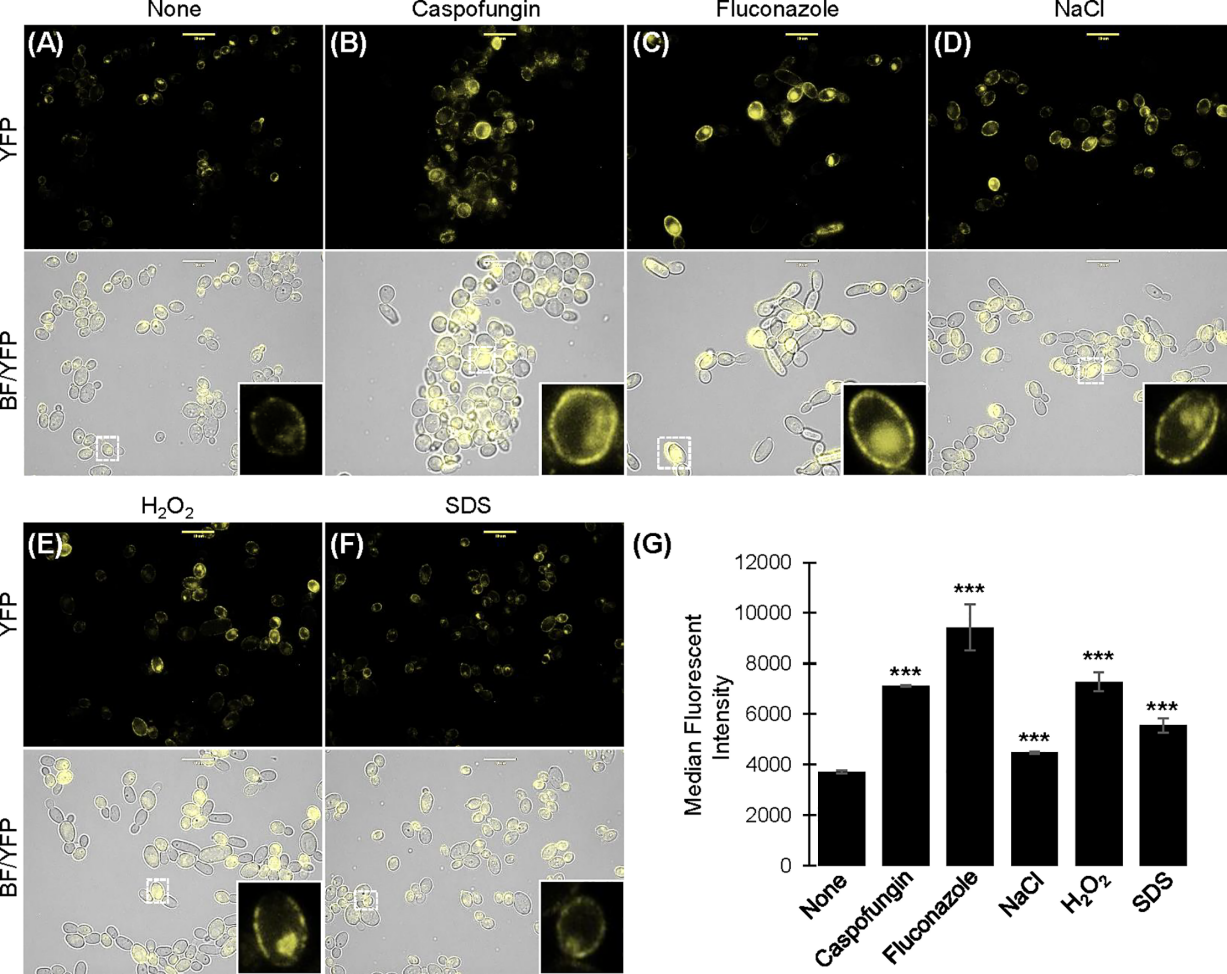
Stress-induced localization of Slp3p. Exponential-phase *SLP3-YFP* cells were incubated in YPD^+uri^ for 30 minutes with the following additives: (A) None, (B) 125 ng/mL caspofungin, (C) 2.5 μg/mL fluconazole, (D) 1.0 M NaCl, (E) 0.17% H_2_O_2_, or (F) 0.08% SDS. Samples were observed under bright-field and fluorescent microscopy. Dashed white boxes shows the cell depicted in the inset image. (G) Fluorescence was quantified by flow cytometry. For each assay, three biological replicates were analyzed. Experiments were repeated at least three times, and data presented represents one representative experiment. ***p < 0.001 when compared to the untreated sample. Approximately 1.0 x 10^4^ cells of each strain were selected for viewing (A-F). Scale bars represent 10 μm.

Cell wall remodeling, fatty acid metabolism, and activation of the oxidative stress response are physiological processes linked to cationic stress [16]. We hypothesized that *SLP3* expression increases in response to diverse types of environmental stress. We examined cellular fluorescence in exponential-phase *SLP3-YFP* cells briefly treated with compounds that induce cell wall stress (caspofungin), plasma membrane stress (fluconazole), or oxidative stress (SDS and H_2_O_2_). Our fluorescent microscopy results show that cellular fluorescence was significantly higher under all stress treatments compared to untreated *SLP3-YFP* cells (Fig 4A–4F). Quantitative results show that the change in fluorescent intensity was the greatest in fluconazole-treated cells, and the least following NaCl treatment when compared against untreated cells (Fig 4G). To support our cellular findings, we examined *SLP3* transcription from 62 gene expression datasets under various physiological and environmental conditions [18]. *SLP3* transcription was reported on 21 datasets, each of which was generated using different array platforms and cell strains. Four datasets showed *SLP3* transcription increased following treatment with oxidative, osmotic, and plasma membrane stress agents, and when cells were grown in high levels of ammonium [39–42]. *SLP3* transcription was reduced when cells were induced to form hyphae, subjected to endoplasmic reticulum stress, or exposed to heat shock [43–45].

**Fig 5:**
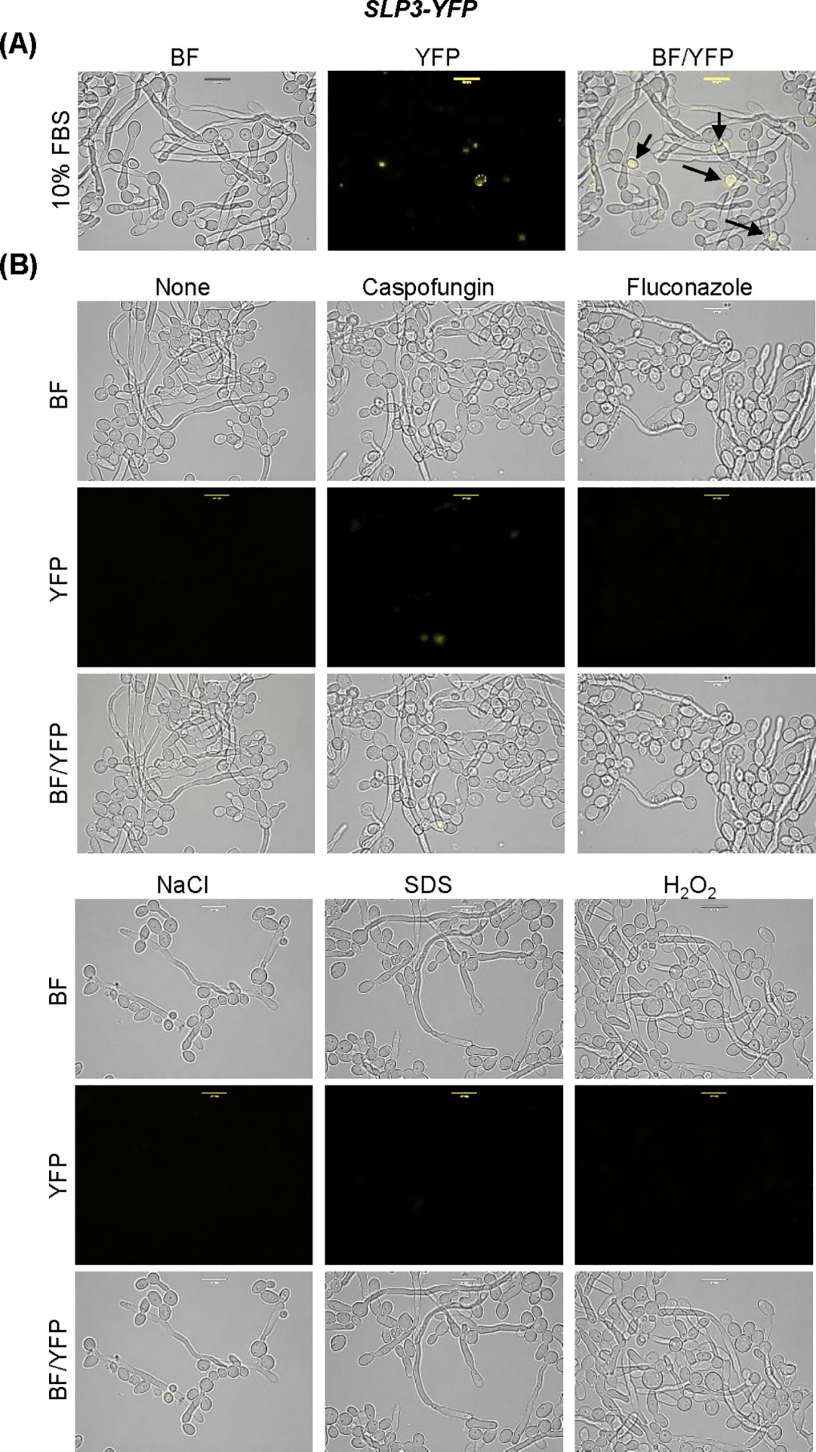
Localization of Slp3p in the yeast-to-hyphae transition. (A) *SLP3-YFP* cells were grown in 10% FBS/YPD^+uri^ medium for 16 hours and observed under bright-field and fluorescent microscopy. Arrows highlight fluorescent yeast-phase cells. (B) Overnight cultured *SLP3-YFP* hyphal cells were standardized in 10% FBS/YPD^+uri^ media and incubated for 45 minutes with the given additives and viewed under bright-field and fluorescent microscopy. The concentration of additives used is as follows: 125 ng/mL caspofungin, 2.5 μg/mL fluconazole, 1.0 M NaCl, 0.17% H_2_O_2_, and 0.08% SDS. For each assay, three biological replicates were analyzed. Experiments were repeated at least three times, and data presented represents one representative experiment. Approximately 1.0 x 10^4^ cells of each strain were selected for viewing. Scale bars represent 10 μm.

We wanted to determine whether Slp3p is present in hyphae, since *SLP3* transcription is significantly reduced in the yeast-to-hyphae transition. We monitored Slp3p-Yfp localization of cells undergoing the yeast-to-hyphae transition. We did not observe fluorescence in pseudohyphal cells or germ tubes on mature hyphae (Fig 5A). In addition, we did not observe Slp3p-Yfp localization in hyphae when cells were grown in the presence of various environmental stress agents (Fig 5B).

The absence of Slp3p in hyphae was unexpected, because stomatins are expressed in filamentous fungi. *A*. *nidulans* StoA is orthologous to Slp3p and localizes to the cortex of hyphal tips [6]. We examined the microarray dataset from the Nantel et al. genome profiling study [45] to determine if the other *C*. *albicans* SPFH family members (*PHB1*, *PHB2*, *PHB12*, *SLP2*) are also repressed in hyphal formation. We found that *SLP3* is the sole SPFH family member repressed in the yeast-to-hyphae transition. Our stress-induced localization findings and genome-wide transcriptional profiling results from other groups demonstrate that *SLP3* is a yeast-phase specific, general stress response gene. Further, the absence of Slp3p in hyphal cells reflects the organism- specific attributes of stomatin proteins in *C*. *albicans* compared to other filamentous fungi.

### Analysis of *SLP3* mutants

**Fig 6:**
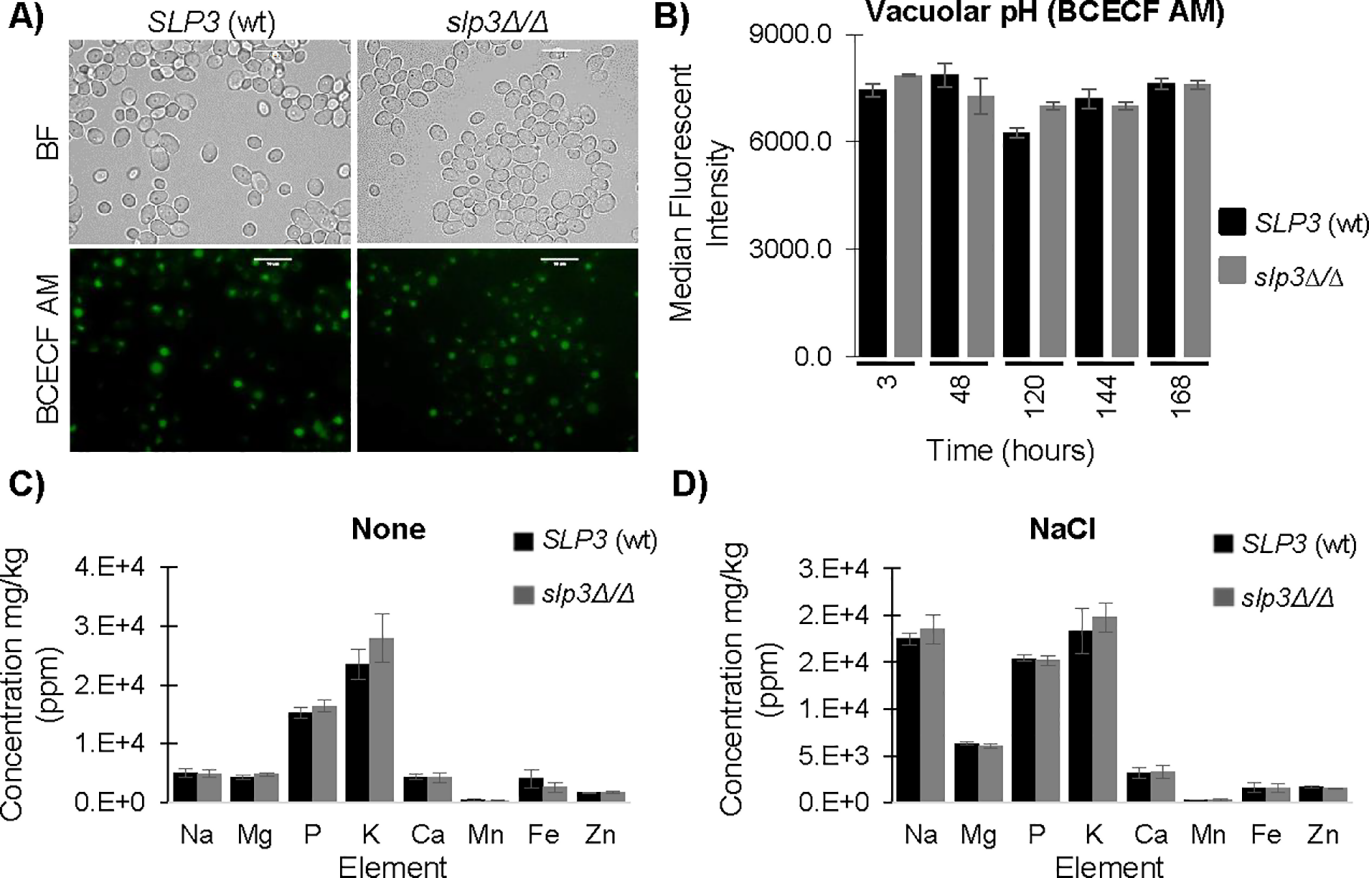
Analysis of intracellular ion levels and vacuolar function in the *slp3Δ/Δ* mutant strain. (A) Exponential-phase samples of wt *SLP3* and homozygous null *slp3Δ/Δ* mutant cells were stained with 10 μM BCECF AM for 40 minutes, and washed before visualizing using bright-field and fluorescence microscopy. Approximately 1.0 x 10^4^ cells of each strain were selected for viewing. (B) Flow cytometry was used to monitor vacuolar acidification at the indicated time points in yeast exponential and stationary phase cells. For each assay, three biological replicates were analyzed. Experiments were repeated at least three times, and data presented represents one representative experiment. Scale bars represent 10 μm. (C) Inductively coupled plasma mass spectrometry analysis was performed on exponential-phase wt control *SLP3* cells and *slp3Δ/Δ* homozygous null mutant cells grown in nutrient YPD^+uri^ medium and (D) following treatment with 1.0 M NaCl for 30 minutes as described in Methods. Mean values were calculated based on values obtained from three biological replicates. Two independent experiments were performed, and data presented represents one representative experiment.

Our expression and localization findings suggested that Slp3p might be required for growth when cells are subjected to stress and that Slp3p may be necessary for vacuolar function. We created a homozygous *slp3Δ/Δ* null mutant strain to explore the function of Slp3p. We did not observe a growth defect under varying environmental conditions, nor did we observe any defects in hyphae formation, cell wall and cytoskeletal structure, and endocytosis (Table 4). Because Slp3p localized to the vacuole, we examined vacuolar structure and function in the *slp3Δ/Δ* null mutant strain. We did not observe any vacuolar structural defects in *slp3Δ/Δ* mutant cells labeled with FM 4–64 (Table 4). We used the fluorescent probe BCECF AM to monitor vacuolar acidification in exponential and stationary phase yeast cells, since several vacuolar functions (protein sorting, ion storage, and enzyme function) require an acidic environment [46]. Similar to our FM 4–64 results, we did not observe a vacuolar acidification defect in the *slp3Δ/Δ* null mutant strain (Table 4 and Fig 6A and 6B).

**Table 4:** Summary of phenotypes in *SLP3* mutant strains.

		Strain	
		*slp3Δ/Δ*	*P*_*TDH3*_*SLP3-YFP*
**Process and Cell Structure**^**a**^	Endocytosis and Vacuole Structure	wt	wt
	Vacuole pH	wt	wt
	Cytoskeleton	wt	wt
	Cell Wall	wt	wt
	Plasma Membrane	wt	wt
**Growth in Solid Media**^**b**^	No Additive	wt	wt
	No Additive (37°C)	wt	wt
	0.04% SDS	wt	Sensitive
	0.08% SDS	wt	Sensitive
	20 μM Calcofluor White	wt	wt
	13 mM Copper Sulfate	wt	wt
	15 mM Copper Sulfate	wt	wt
	125 ng/μL Caspofungin	wt	wt
	50 ng/μ. L Caspofungin	wt	wt
	10% Glucose	wt	wt
	2.0 M NaCl	wt	wt
	1.5 M NaCl	wt	wt
	1.0 M NaCl	wt	wt
	1.0 M KCl	wt	wt
	10 mM Caffeine	wt	wt
	1.5 M Sorbitol	wt	wt
**Growth in Liquid Media**^**c**^	No Additive	wt	wt
	0.17% H_2_O_2_	wt	Viability Reduced
	0.08% SDS	wt	Viability Reduced
	10% FBS	wt	Viability Reduced
	Spider	wt	Viability Reduced

^a^These rows list the physiological processess and cell structural features examined in the wt *SLP3* strain and isogenic *slp3Δ/Δ* mutant and *P*_*TDH3*_*SLP3-YFP* over-expressing strains. wt is used to denote identical phenotpyes in the mutant and over-expression strains compared to the wt strain. Exponential phase yeast cells were collected, stained, and visualized under bright-field and fluorescent microscopy as described in Methods. The following labels were used in each assay: 160 μM FM 4–64 (endocytosis and vacuole structure), 10 μM BCECF AM (vacuole pH), 100 μg/mL calcofluor white (cell wall), 6.6 μM phalloidin rhodamine and 150 nM Tubulin tracker green (cytoskeleton) and 200 μg/mL Filipin III (plasma membrane). For each assay, three biological replicates were analyzed, and experiments were repeated at least three times.

^b^These rows list the growth phenotypes of *SLP3* strains on solid YPD^+uri^ nutrient plates (no additive) and YPD^+uri^ plates supplemented with the indicated additive. Cells from overnight cultures were collected, serially diluted, and and spotted onto the designated plates. The term sensitive denotes *P*_*TDH3*_*SLP3-YFP* over-expressing cells that did not grow on plates following 1–3 days growth compared to the *SLP3* wt strain. Plates were incubated at 30°C, unless otherwise noted on the table. For each assay, three biological replicates were analyzed.

^c^These rows list the growth phenotypes of *SLP3* strains in YPD^+uri^ liquid medium (no additive) and YPD^+uri^ media supplemented with the indicated additive. Cell growth was monitored in a microplate reader for assays using H_2_O_2_ and SDS. For each assay, three biological replicates were analyzed and experiments were repeated two times. FBS and Spider media were used to examine the yeast-to-hyphae transition, and cells were visualized under the microscope. The term viability reduced is used to denote *P*_*TDH3*_*SLP3-YFP* over-expressing yeast or hyphal cells that stained with Propidium Iodide compared to wt *SLP3* cells following incubation in their respective media.

We tested the hypothesis that Slp3p is required for ion homeostasis in *C*. *albicans*, because stomatins modulate ion channel activity during mechanosensation in mammals [10]. Furthermore, the vacuole is required for ion storage [38]. We performed inductively coupled plasma mass spectrometry to measure intracellular ion levels from exponential-phase cultures under normal growth conditions and following salt stress. Under both conditions, the concentration of individual elements varied; however, there were no significant differences between the wt *SLP3* and *slp3Δ/Δ* mutant strains (Fig 6C and 6D). Similar results were observed in parallel experiments using stationary-phase cells (data not shown).

Human SLP-2 was proposed to be a scaffold for mitochondrial proteins –°a role where functional redundancy is common [47]. The absence of a *slp3Δ/Δ* null mutant phenotype suggests that Slp3p may be functionally complemented by another SPFH family protein or uncharacterized protein. *C*. *albicans* Slp2p and Slp3p share limited sequence homology (31% identity, 53% similarity, (S1 Fig and Table 3)), and the function of Slp2p is unknown. We observed a stark difference in transcriptional activity under stress. We did not detect any changes in *SLP2* transcription in the *slp3Δ/Δ* mutant strain under normal growth conditions or following cationic stress using RT-qPCR (data not shown). In addition, *SLP2* transcription was unchanged following multiple stress conditions that significantly modulate *SLP3* transcription [39–42], and Slp2p was not found in the plasma membrane [17]. Although these observations imply that *SLP2* and *SLP3* have divergent functions, we cannot rule out the possibility that functional redundancy is operative on a post-translational level, since *SLP2* is currently uncharacterized.

**Fig 7:**
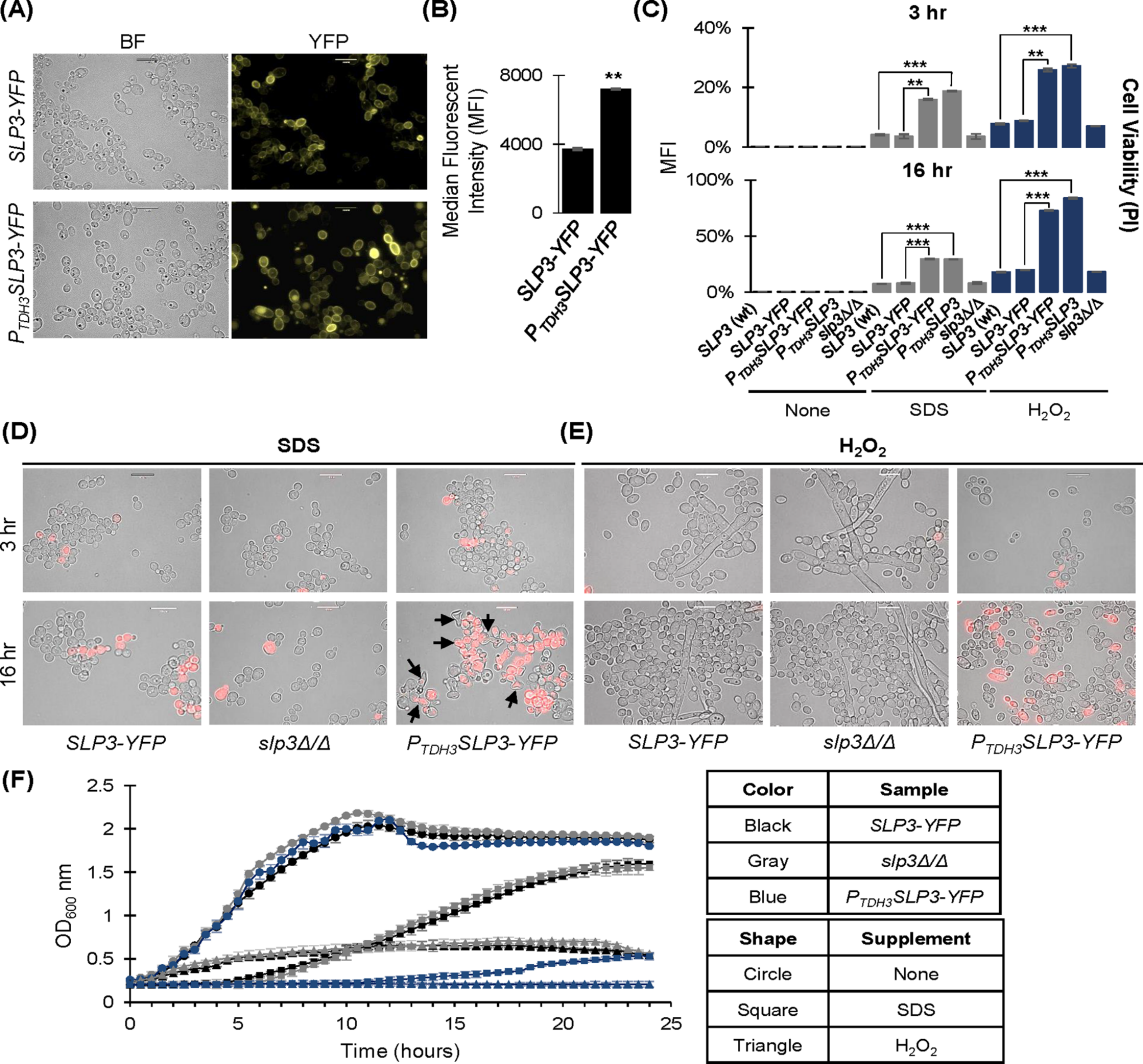
Phenotype of *SLP3* over-expression. (A) Exponential-phase samples of *P*_*TDH3*_*SLP3-YFP* over-expressing cells and *SLP3-YFP* cells were examined under bright-field and fluorescent microscopy. (B) Samples described in (A) were quantified by flow cytometry. (C-E) Overnight yeast cultures were diluted into YPD^+uri^ supplemented with the indicated additive and grown for 3 or 16 hours. Cells were treated with PI and examined using bright-field and fluorescent microscopy. Cells were quantified by flow cytometry. (F) Stationary phase cultures were standardized in the appropriate media, and growth kinetics were analyzed for 24 hours in the presence and absence of the indicated additive. For each assay, three biological replicates were analyzed. Experiments were repeated at least three times, and data presented represents one representative experiment. *p < 0.05, **p < 0.01, ***p < 0.001 when compared to the isogenic parental strain. Approximately 1.0 x 10^4^ cells of each strain were selected for viewing (A, D-E). Scale bars represent 10 μm.

Novel functional roles of stomatins have been implicated in over-expression analysis. In humans, over-expression of STOM and SLP-1 implicated roles in placental trophoblast differentiation and protein trafficking, respectively [31, 48]. Also, SLP-2 over-expression was associated with several clinicopathological features in thyroid cancer patients [49]. Therefore, we constructed a *P*_*TDH3*_*SLP3-YFP* over-expressing strain as an alternative approach to study Slp3p function. Over-expression did not alter the localization of Slp3p, and cellular fluorescence was approximately 2.0 times greater compared to the *SLP3-YFP* control strain (Fig 7A and 7B). Strikingly, we found that growth and cell viability were significantly reduced when *P*_*TDH3*_*SLP3-YFP* over-expressing cells were treated with the oxidative stress agents SDS and H_2_O_2_ (Fig 7C–7F and Table 4). Growth inhibition was apparent at 16 hours of exposure to oxidative stress compared to 3 hours. Cell morphology was abnormal in SDS-treated *P*_*TDH3*_*SLP3-YFP* over-expressing cells. Many cells appeared to be ruptured or misshapen and formed cellular aggregates (Fig 7D). At 16 hours, 30% of *P*_*TDH3*_*SLP3-YFP* over-expressing cells were stained with PI, which marks cells with compromised membrane integrity. Only 8% of the *slp3Δ/Δ* null mutant and 7% wt *SLP3*, and *SLP3-YFP* cells were stained with PI (Fig 7C). For H_2_O_2_-treated *P*_*TDH3*_*SLP3-YFP* over-expressing cells, the yeast cell morphology was similar to the *SLP3-YFP* and *slp3Δ/Δ* mutant strains; however, the over-expressing strain failed to form filaments (Fig 7E). Similar to the SDS-treated cells, 72% of *P*_*TDH3*_*SLP3-YFP* over-expressing cells were stained with PI while only 18% of the *slp3Δ/Δ* mutant and wt *SLP3* cells and 20% of the *SLP3-YFP* cells were stained with PI following 16 hours treatment (Fig 7C).

The growth phenotype of the *P*_*TDH3*_*SLP3-YFP* over-expressing strain was exclusive to oxidative stress, since growth was similar to the *SLP3-YFP* parental strain and untagged isogenic wt *SLP3* strain under all other conditions examined (Table 3). In addition, we did not identify any defects in vacuolar structure and function, cell wall and cytoskeletal structure, and endocytosis (Table 3). We considered the possibility that the *P*_*TDH3*_*SLP3-YFP* oxidant growth defect was due to YFP over-expression, because fluorescent tags may interfere with the expression, localization, or function of a protein of interest [50]. We constructed a *P*_*TDH3*_*SLP3* over-expressing strain without YFP and assayed viability under oxidative stress. Our results show that the H_2_O_2_ and SDS viability phenotypes were similar in the *P*_*TDH3*_*SLP3-YFP* and *P*_*TDH3*_*SLP3* over-expressing strains (Fig 7C). Thus, over-expression of *SLP3* is lethal when cells are subjected to oxidative stress.

### Slp3p over-expression initiates apoptotic-like death

**Fig 8:**
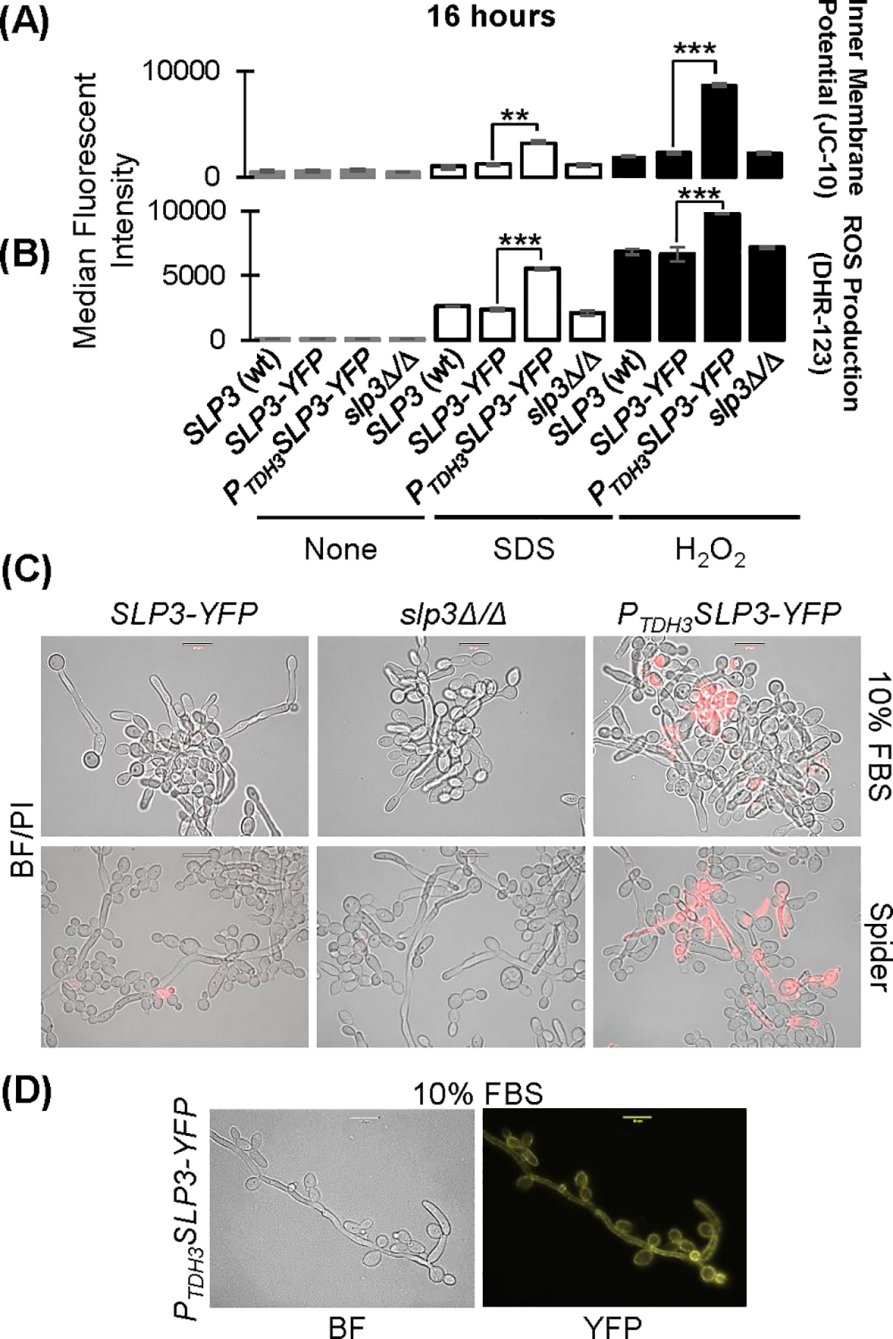
Analysis of apoptosis in *SLP3* over-expressing cells. (A) Mitochondrial membrane potential was measured in overnight cultured cells following exposure with the given additives for 16 hours. Samples were labeled using JC-10 and quantified using flow cytometry. (B) Intracellular ROS production was studied in yeast cells following incubation with the indicated additives for 16 hours. Samples were labeled with DHR-123 and quantified using flow cytometry. Gray bars represent untreated samples; white bars represent samples treated with 0.08% SDS, and black bars represent samples treated with 0.17% H_2_O_2_. Similar findings were observed in samples treated for 3 hours. (C) *P*_*TDH3*_*SLP3-YFP* cells were grown in 10% FBS/YPD^+uri^ and Spider media to observe the yeast-to-hyphae transition and stained with PI to assess viability. Samples were visualized using bright-field and fluorescence microscopy. (D) Slp3p puncta were visualized in the plasma membrane of yeast-phase cells and hyphal germ tube using bright-field and fluorescent microscopy. For each assay, three biological replicates were analyzed. Experiments were repeated at least three times, and data presented represents one representative experiment. *p < 0.05, **p < 0.01, ***p < 0.001 when compared to the isogenic parental strain. Approximately 1.0 x 10^4^ cells of each strain were selected for viewing. Scale bars represent 10 μm.

Oxidative stress induces apoptotic death in *C*. *albicans* [51]. Phosphatidylserine exclusion (indicated by PI staining), reactive oxygen species (ROS) production, and mitochondrial inner membrane depolarization are major cellular events associated with apoptosis [52]. Accordingly, we tested whether the hypersensitivity of *P*_*TDH3*_*SLP3-YFP* over-expressing cells to oxidants coupled with the increased uptake of PI was a consequence of cells undergoing apoptosis. We assayed ROS production (DHR-123 treatment), and mitochondrial inner membrane potential (JC-10 labeling) in *P*_*TDH3*_*SLP3-YFP* over-expressing cells after 16 hours of oxidative stress exposure. Consistent with cells undergoing apoptosis, the percentage of *P*_*TDH3*_*SLP3-YFP* over-expressing cells with SDS- or H_2_O_2_-induced depolarized inner mitochondrial membranes was statistically greater compared to the *slp3Δ/Δ* null mutant and wt *SLP3* strains after 16 hours exposure (Fig 8A and S4 Fig). ROS levels were statistically greater in the *P*_*TDH3*_*SLP3-YFP* over-expressing strain compared to the wt *SLP3*, *SLP3-YFP*, and *slp3Δ/Δ* mutant strains after 16 hours exposure (Fig 8B). We did not detect any apoptotic activity in over-expressing cells in the absence of stress.

The mitochondrion is critical in mediating homeostasis to oxidative stress. Human SLP-2 interacts synergistically with prohibitins at the inner mitochondrial membrane to promote mitochondrial stability [53]. We did not identify a mitochondrial targeting sequence in Slp3p (S1 Table) or observe Slp3p-Yfp mitochondrial localization following oxidative stress or under any other condition that we screened (Figs 2–4). Therefore, the mechanism by which *SLP3* over-expression causes mitochondrial depolarization may be indirect. Nevertheless, *SLP3* over-expression leads to inner mitochondrial membrane depolarization, phosphatidylserine exclusion, and increased levels of ROS, specifically when cells are subjected to prolonged oxidative stress, ultimately leading to apoptotic-like death.

H_2_O_2_ exposure initiates filamentation in *C*. *albicans*. H_2_O_2_-induced filaments are presumed to be indicative of a disrupted cell cycle and are morphologically distinct (a state called hyperpolarized buds) compared to filaments generated in nutrient filamentation media at 37^°^C [51]. As seen in Fig 7E, filamentous cells are present in the *SLP3-YFP* strain following 3 and 16 hours of H_2_O_2_ exposure, but were absent in the *P*_*TDH3*_*SLP3-YFP* over-expressing strain. SDS-treated *SLP3-YFP* cells did not form filaments (Fig 7D), which may seem contradictory to our observations using H_2_O_2._ However, while SDS kills *C*. *albicans* mainly by inducing oxidative stress, it also inhibits filamentation [54].

We wanted to determine whether the filamentation defect in the *P*_*TDH3*_*SLP3-YFP* over-expressing strain was specific to H_2_O_2_ exposure or a general phenotype associated with Slp3p over-expression. We compared filamentation of the over-expressing strain to the wt strain after 3 and 16 hours incubation in serum. In contrast to our observations with H_2_O_2_-treated *P*_*TDH3*_*SLP3-YFP* over-expressing cells, we did not observe any differences in hyphal formation or hyphal morphology after 3 hours (data not shown) and 16 hours of growth in serum or Spider medium (Fig 8C). Slp3p-Yfp puncta were present throughout the hyphal germ tube, confirming Slp3p plasma membrane localization (Fig 8D). On the other hand, several *P*_*TDH3*_*SLP3-YFP* over-expressing cells grown in serum for 16 hours were PI positive compared to *SLP3-YFP* and *slp3Δ/Δ* null mutant cells, indicating that hyphal formation was lethal in the absence of environmental stress (Fig 8C). Taken together, our findings show that Slp3p over-expression inhibits filamentation under H_2_O_2_ stress, but compromises cellular integrity when cells are grown in filamentation medium.

In summary, our work is the first to provide functional insight and fully characterize the localization of a stomatin family member in *C*. *albicans*. Our primary sequence analyses showed that the SPFH domain of Slp3p is homologous to mammalian and nematode stomatins, while N- and C-terminal flanking sequences are divergent. Further, we identified putative sites for plasma membrane insertion and vacuolar targeting. We argue that *SLP3* is a yeast-phase specific stress response gene, because *SLP3* expression and cell surface localization increases following exposure to diverse cellular perturbants in yeast cells and not in hyphal cells. The lack of phenotype in a *slp3Δ/Δ* homozygous null mutant implies stomatin function may be redundant in *C*. *albicans*. Conversely, Slp3p over-expression triggers apoptotic-like death specifically following prolonged exposure to oxidative stress. This lethality may be due in part to the inability to form filaments. We note the limitation of these phenotypic observations, since Slp3p over-expression may indirectly alter molecular processes that govern apoptosis and filamentation. Nevertheless, our results are consistent with *SLP3* having a positive role in response to oxidative stress in yeast phase cells.

## Supporting information

S1 Fig Primary sequence alignment of Slp3p compared to mammalian, nematode, and fungal SPFH proteins. Alignments 1 and 2: Residues highlighted in black are identical while those highlighted in gray are similar between SPFH family members. Residues highlighted within red boxes constitute endosome/lysosome targeting sequences. Residues within the green box constitute the membrane hairpin region of mammalian and nematode stomatins. The conserved proline residue within this region is indicated with a black triangle. The N-terminal region consists of residues that lie before the membrane hairpin region. Residues highlighted within dark blue boxes constitute the SPFH domain. Residues highlighted within orange boxes constitute the SCP-2 domain of HsSTOML1. The C-terminal region consists of residues that lie after the SPFH domain. Potential palmitoylation sites are highlighted with an asterisk. Lysine residues labeled with an orange “K” are potential SUMOylation sites. Gaps are denoted with a hyphen. Ca: *Candida albicans*. Hs: *Homo sapiens*. Ms: *Mus musculus*. Ce: *Caenorhabditis elegans*. An: *Aspergillus nidulans*.
(PPTX)

S2 Fig Vacuolar localization of Slp3p. Overnight cultured *SLP3-YFP* cells were standardized to an OD_600nm_ of 0.2 in YPD^+uri^ and incubated at 30°C. At (A) 3, (B) 17, and (C-D) 24 hours, samples were viewed using fluorescent microscopy. Cells in (D) were labeled with 160 μM FM 4–64. Dashed white boxes show the cells depicted in the inset. For each assay, three biological replicates were analyzed. Experiments were repeated at least three times, and data presented represents one representative experiment. Approximately 1.0 x 10^4^ cells of each strain were selected for viewing. Scale bars represent 10 μm.
(TIFF)

S3 Fig Salt-induced localization of Slp3p. Exponential-phase *SLP3-YFP* cells were treated with the given additives for 30 minutes and visualized with bright-field and fluorescent microscopy. Concentrations of additives used are as follows: 1.0 M NaCl, 1.0 M KCl, 1.0 M MgCl_2_, 10 mM ZnCl_2_, 1 mM FeCl_3_, 0.6 M CaCl_2_, 0.6 M LiCl, and 50 mM CuCl_2_. Water served as the negative control. For each assay, three biological replicates were analyzed. Experiments were repeated at least three times, and data presented represents one representative experiment. Approximately 1.0 x 10^4^ cells were selected for viewing. Scale bars represent 10 μm.
(TIF)

S4 Fig Mitochondrial depolarization in Slp3p over-expressing cells. Overnight cultured *SLP3-YFP* cells, *slp3Δ/Δ* homozygous null mutant cells, and *P*_*TDH3*_*SLP3-YFP* cells were standardized in YPD^+uri^ media or YPD^+uri^ media supplemented with 0.08% SDS and incubated for 16 hours at 30°C. Samples were prepared and stained with 1X JC-10 according to the manufacturer’s protocol. Cells were visualized using bright-field and fluorescent microscopy. Cells with intact mitochondria fluoresce red, and cells with depolarized mitochondria fluoresce green. For each assay, three biological replicates were analyzed. Experiments were repeated at least three times, and data presented represents one representative experiment. Approximately 1.0 x 10^4^ cells of each strain were selected for viewing. Scale bars represent 10 μm.
(TIFF)

S1 Table Table includes results of Slp3p primary sequence analyses. (XLSX)

S1 Appendix Appendix includes raw flow cytometry data of Slp3p growth-phase dependent localization experiments. (XLSX)
