# Supplementary figures and images for: The Candida albicans stress response gene Stomatin-Like Protein 3 is implicated in ROS-induced apoptotic-like death of yeast phase cells
Source: PLoS One. 2018 Feb 1;13(2):e0192250. doi: 10.1371/journal.pone.0192250 (PMC5794166; doi:10.1371/journal.pone.0192250)

## Slide 1
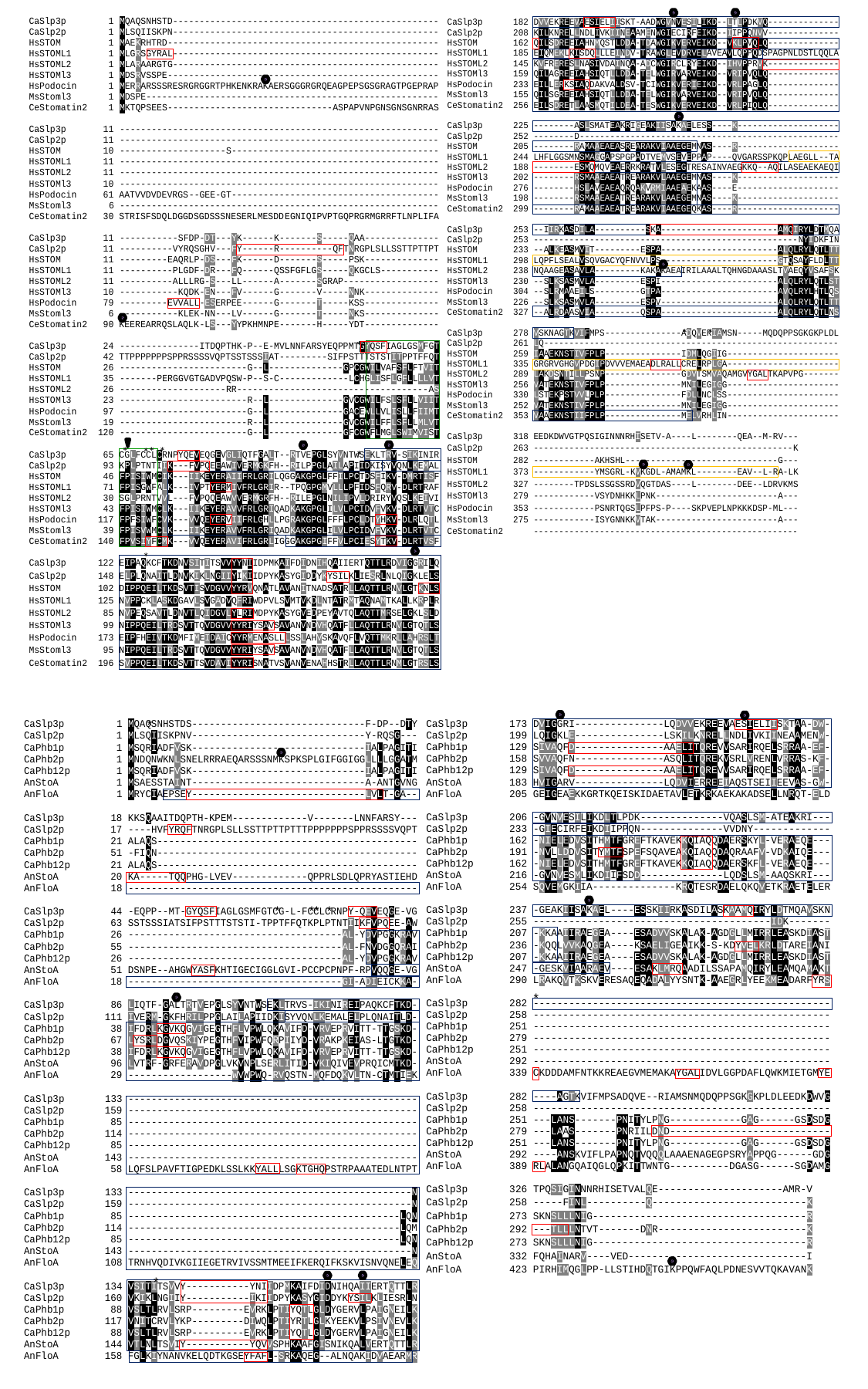

K
K
K
K
K
K
*
*
*
*
*
*
*
*
K
K
K
K
*
K
*
K
K
K
*
*
*
*
K
*
K
K
*
K
K

Supplement: S1 Fig — Alignments 1 and 2: Residues highlighted in black are identical while those highlighted in gray are similar between SPFH family members. Residues highlighted within red boxes constitute endosome/lysosome targeting sequences. Residues within the green box constitute the membrane hairpin region of mammalian and nematode stomatins. The conserved proline residue within this region is indicated with a black triangle. The N-terminal region consists of residues that lie before the membrane hairpin region. Residues highlighted within dark blue boxes constitute the SPFH domain. Residues highlighted within orange boxes constitute the SCP-2 domain of HsSTOML1. The C-terminal region consists of residues that lie after the SPFH domain. Potential palmitoylation sites are highlighted with an asterisk. Lysine residues labeled with an orange “K” are potential SUMOylation sites. Gaps are denoted with a hyphen. Ca: Candida albicans. Hs: Homo sapiens. Ms: Mus musculus. Ce: Caenorhabditis elegans. An: Aspergillus nidulans. (PPTX) [file pone.0192250.s001.pptx]

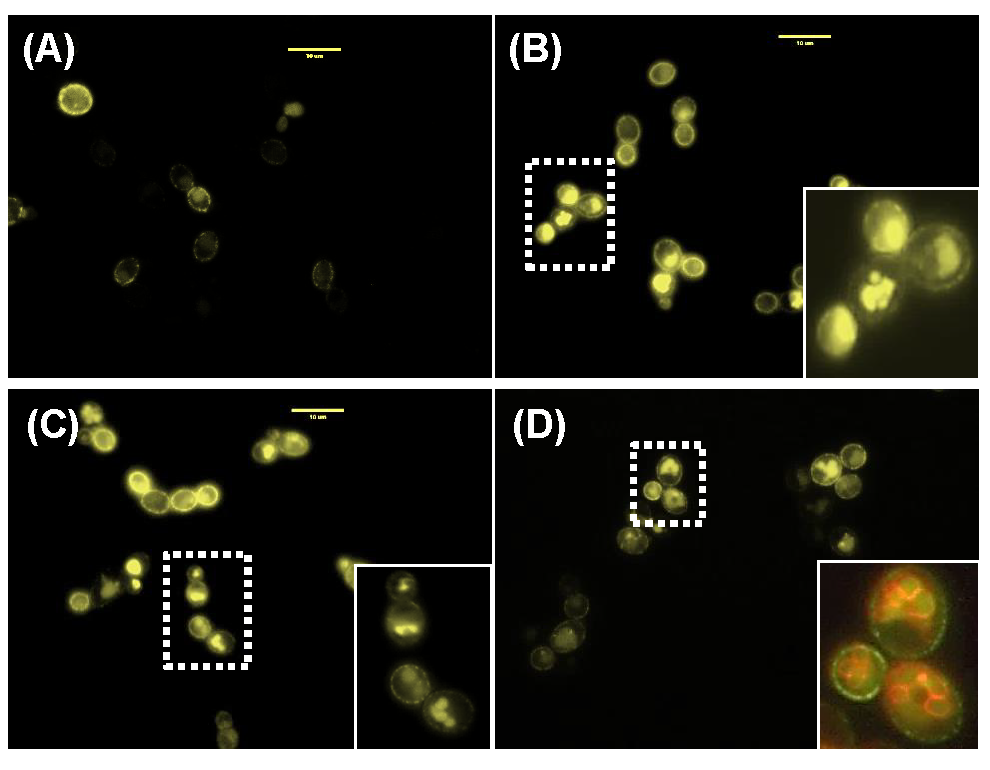

Supplement: S2 Fig — Overnight cultured SLP3-YFP cells were standardized to an OD600nm of 0.2 in YPD+uri and incubated at 30°C. At (A) 3, (B) 17, and (C-D) 24 hours, samples were viewed using fluorescent microscopy. Cells in (D) were labeled with 160 μM FM 4–64. Dashed white boxes show the cells depicted in the inset. For each assay, three biological replicates were analyzed. Experiments were repeated at least three times, and data presented represents one representative experiment. Approximately 1.0 x 10^4^ cells of each strain were selected for viewing. Scale bars represent 10 μm. (TIFF) [file pone.0192250.s002.tiff]

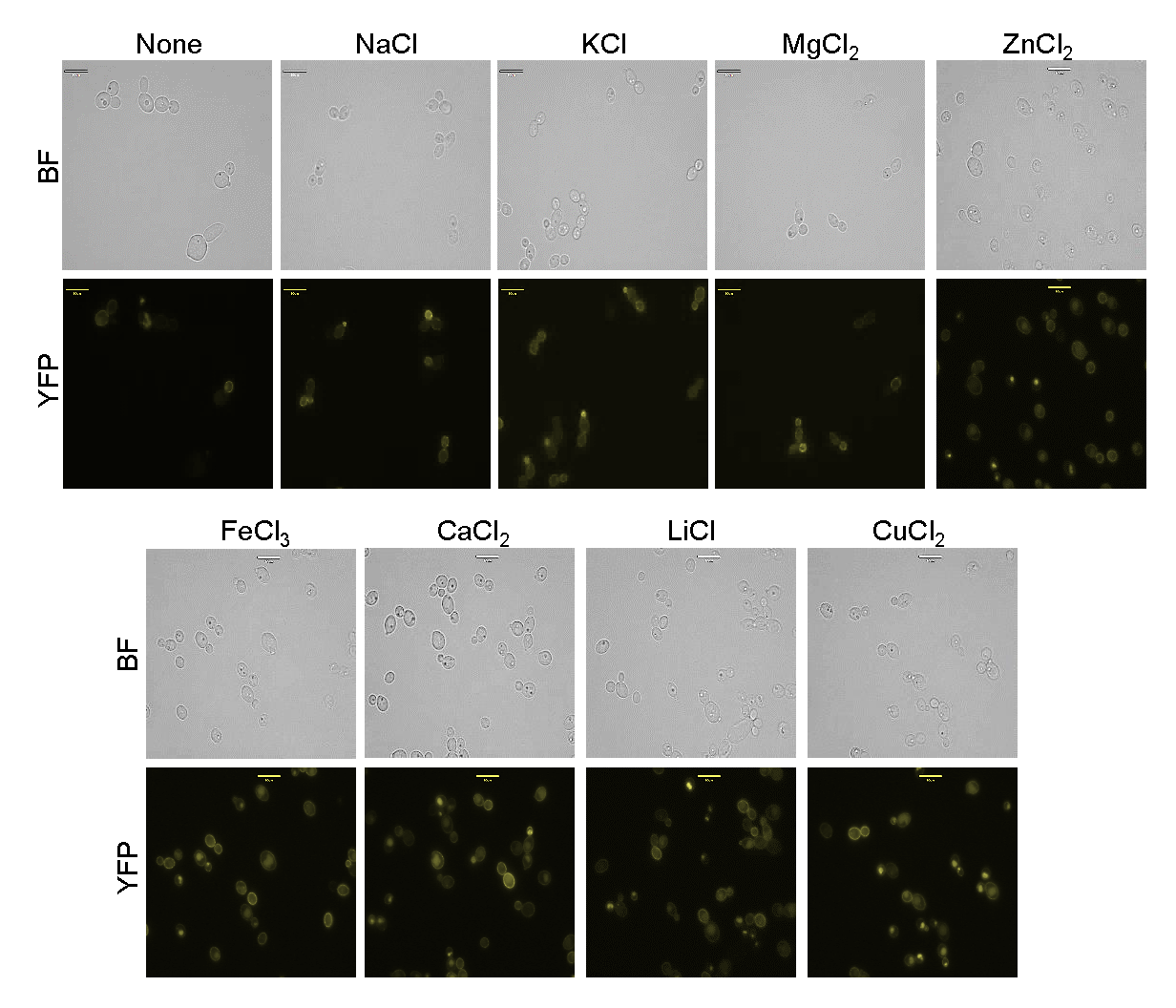

Supplement: S3 Fig — Exponential-phase SLP3-YFP cells were treated with the given additives for 30 minutes and visualized with bright-field and fluorescent microscopy. Concentrations of additives used are as follows: 1.0 M NaCl, 1.0 M KCl, 1.0 M MgCl2, 10 mM ZnCl2, 1 mM FeCl3, 0.6 M CaCl2, 0.6 M LiCl, and 50 mM CuCl2. Water served as the negative control. For each assay, three biological replicates were analyzed. Experiments were repeated at least three times, and data presented represents one representative experiment. Approximately 1.0 x 10^4^ cells were selected for viewing. Scale bars represent 10 μm. (TIF) [file pone.0192250.s003.tif]

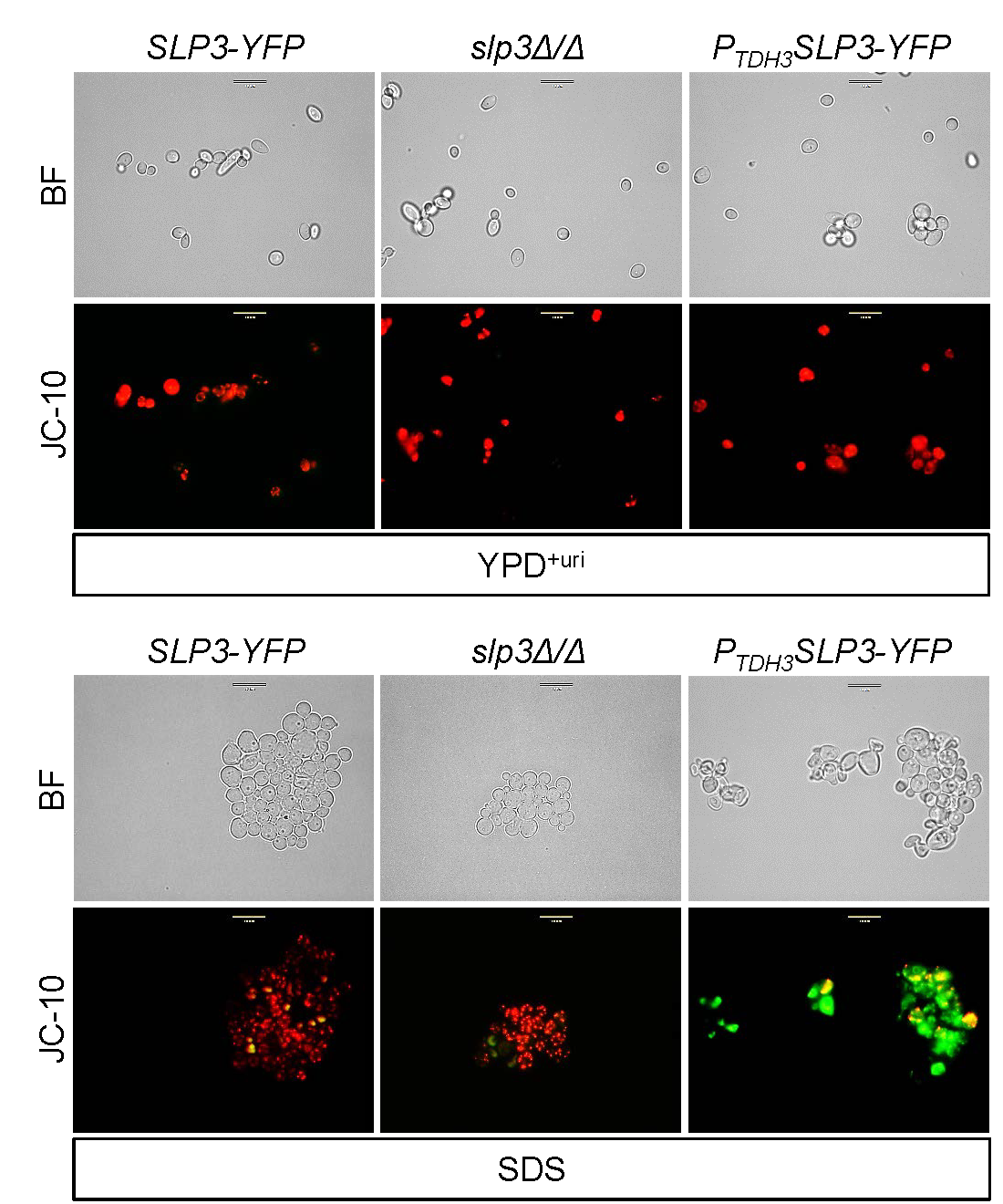

Supplement: S4 Fig — Overnight cultured SLP3-YFP cells, slp3Δ/Δ homozygous null mutant cells, and PTDH3SLP3-YFP cells were standardized in YPD+uri media or YPD+uri media supplemented with 0.08% SDS and incubated for 16 hours at 30°C. Samples were prepared and stained with 1X JC-10 according to the manufacturer’s protocol. Cells were visualized using bright-field and fluorescent microscopy. Cells with intact mitochondria fluoresce red, and cells with depolarized mitochondria fluoresce green. For each assay, three biological replicates were analyzed. Experiments were repeated at least three times, and data presented represents one representative experiment. Approximately 1.0 x 10^4^ cells of each strain were selected for viewing. Scale bars represent 10 μm. (TIFF) [file pone.0192250.s004.tiff]
